# A cross-sectional, multicenter, observational study to assess the prophylaxis of venous thromboembolism in Lebanese and Jordanian hospitals

**DOI:** 10.1186/s12959-021-00261-2

**Published:** 2021-02-10

**Authors:** Imad Hajj, Mahmoud AL-Masri, Kaldoun Bashaireh, Mohammed Bani Hani, Shadi Hamouri, Joe Khouzami, Nisrine Sabra, Chahine Fadel

**Affiliations:** 1 Department of Surgery, Saint George Hospital University Medical Center, Ashrafiyeh, Beirut, Lebanon; 2 Department of Surgery, King Hussein Cancer Center, PO Box 1269, Al-Jubeiha, Amman, 11941 Jordan; 3 Department of Special Surgery, Faculty of Medicine, Jordan University of Science and Technology, Ar-Ramtha, Jordan; 4 Department of General Surgery and Urology, Faculty of Medicine, Jordan University of Science and Technology, King Abdallah University Hospital, Ar-Ramtha, Jordan; 5 Sanofi, Beirut, Lebanon

**Keywords:** Venous thromboembolism, VTE prophylaxis, ACCP guideline

## Abstract

**Background:**

There is a growing body of evidence showing substantial underuse of appropriate venous thromboembolism (VTE) prophylaxis in patients at risk. In the present study, our goal was to assess the current practices in the use rate of VTE prophylaxis among hospitalized patients in Jordan and Lebanon.

**Methods:**

A cross-sectional, multicenter, observational study was conducted on 40 centers across Lebanon and Jordan. We included patients who were admitted to the participating hospitals for the treatment of a serious medical or surgical illness. The patients’ records were screened for the fulfillment of inclusion/exclusion criteria during a single assessment visit. The proportion of medical and surgical patients who were at risk of VTE and the thrombo-prophylactic measures employed by physicians for these patients were assessed according to the American College of Chest Physicians (ACCP 2016) guidelines.

**Results:**

The present study included 704 patients (400 from Jordan and 304 from Lebanon) with a mean age of 54.9 ± 17.5 years. Almost 59% of the patients received prophylaxis treatment in form of pharmacological anticoagulant prophylaxis and/or mechanical prophylaxis. Low molecular weight heparin was the most commonly used anticoagulant for VTE prophylaxis in 366 out of the total 704 (51.9%) patients in the analysis cohort. Two hundred and sixteen patients (52, 95% confidence interval [47.1–56.9%]) received appropriate prophylactic agents out of 415 patients who were eligible for prophylaxis according to the ACCP 2016 guidelines. On the other hand, 199 (72.1, 95% confidence interval [66.4–77.3%) patients received prophylaxis out of 276 ineligible patients. The rate of compliance to guidelines showed wide variations according to the type of hospital, specialty, and the patients’ age. The multivariate logistic regression analysis showed that only age was a significant predictor of appropriate VTE prophylaxis (odds ratio [OR] 1.05, *P* < 0.001).

**Conclusion:**

The rates of the appropriate use of VTE prophylaxis are low in Lebanon and Jordan. There is a lack of compliance to guidelines for VTE prophylaxis use for hospitalized patients in both countries.

**Supplementary Information:**

The online version contains supplementary material available at 10.1186/s12959-021-00261-2.

## Introduction

Venous thromboembolism (VTE) is a life-threatening disorder and a major cause of morbidities and mortality among hospitalized patients [1]. The condition is characterized by the development of thrombosis of deep veins of the leg or pelvis (DVT) that propagate to the pulmonary circulation leading to pulmonary embolism (PE) [2]. According to previous epidemiological studies, VTE is the third most common cardiovascular disease with reported incidence rates of 130 and 100 per 100,000 persons every year for men and women, respectively [3]. Old age, obesity, prolonged immobility, acute heart failure, malignancy, hyperestrogenemia, and genetic susceptibility are major risk factors for VTE development [4]. Hospitalized patients are at increased risks of VTE due to the presence of multiple risk factors that are usually cumulative [5]. Despite that VTE has been previously described as a complication of major surgery, recently published evidence shows that hospitalized patients with a medical illness have comparable risks of VTE to that of the patients undergoing major surgery [6]. Previous reports demonstrated that up to 20% of hospitalized medical patients are expected to develop VTE during hospital stay [7]. VTE is a major cause of mortality in hospitalized patients as well; up to 10% of fatality cases during hospitalization were attributed to VTE in autopsy-based studies [8]. Therefore, effective VTE prophylaxis among hospitalized at-risk patients, either surgical or medical, is critical in improving patients’ outcomes and survival [9].

Anticoagulants represent the cornerstone for VTE prophylaxis among hospitalized patients [10]. Historically, unfractionated heparin (UFH) was the anticoagulant of choice for VTE prophylaxis; however, the current body of evidence shows that low molecular weight heparin (LMWH) has a comparable efficacy profile, with more favorable safety data than UFH [11]. The American College of Chest Physicians (ACCP 2012) guidelines recommend using prophylactic anticoagulants in cases with a high risk of VTE [12]. They recommend using parenteral LMWH or fondaparinux as a first choice for VTE prophylaxis over IV UFH in the cases of acute deep venous thrombosis (DVT) or pulmonary embolism (PE). They also recommend using thrombolytic therapy in case of PE with hypotension. The guidelines also stated that, in cases of surgically provoked PE or DVT, 3 months of anticoagulant therapy is recommended. Also in the case of PE or DVT that is associated with active cancer, they recommend using LMWH therapy over vitamin K antagonist (warfarin). For extensive superficial vein thrombosis, they recommend a prophylactic therapy with fondaparinux or LMWH instead of no anticoagulants use, and they recommend fondaparinux over LMWH. Other treatment options as compression stockings were recommended to prevent post-thrombotic syndrome. Therefore, LMWH and fondaparinux are currently the first-line prophylaxis options in hospitalized patients [13]. Although multiple international guidelines recommend the use of VTE prophylaxis for at-risk hospitalized patients, the rates of prophylaxis strategies implementation are not satisfied in many regions [9]. The ENDORSE study reported that less than 40% of hospitalized medical patients receiving appropriate prophylaxis, with great variations in prophylaxis use between countries, regions, and hospitals [7].

The burden of VTE is presumably high among hospitalized patients from the Middle East region [14], as many countries from the region are listed among the top ten countries with the highest rate of non-communicable diseases, such as obesity, hypertension, and diabetes [15, 16]. In the AVAIL ME study [14], the rate of patients at a very high-risk for VTE was 40.9% in Iran, 32.7% in Jordan, 27.7% in Saudi Arabia, and 30,7 in Lebanon compared to only 16.7% in Azerbaijan. In the united states of America (USA), VTE is considered a major health problem as nearly 2 million new cases are reported every year with over 600,000 deaths from PE [17]. According to recent reports, the overall compliance with international guidelines concerning VTE prophylaxis was nearly 38% in the Middle Eastern countries, with wide geographical disparity [14]. In comparison to the countries included in the ENDORSE study [7]; the compliance to VTE prophylaxis guidelines was 68% in the USA, 54% in India, 55% in Kuwait, 81% in Germany, 60% in France, 34% in Egypt, 50% in Algeria, and 31% in Argentina [18]. However, the data on the degree of compliance with the current antithrombotic guidelines in the Middle East region is still scarce [19, 20]. Therefore, in this study, we aimed to assess the current practices of VTE prophylaxis among hospitalized patients in Jordan and Lebanon.

## Patients and methods

We followed the STROBE (Strengthening the Reporting of Observational Studies in Epidemiology Statement) guidelines (Supplementary file no.1) during the preparation of this manuscript [21]. The present study runs in concordance with the principles of the declaration of Helsinki and applicable local regulatory laws. Written informed consent was obtained from every eligible patient, or their relatives, prior to the study’s enrollment.

### Study design and setting

We conducted a multicenter, observational, cross-sectional study across Lebanon and Jordan hospitals through the period from October 2017 to October 2018. Participating physicians from 40 centers across both counties were randomly selected and asked to recruit eligible patients consecutively. The selection process of the participating physicians was stratified according to the type of hospital, specialties, and geographical area.

### Participants

Adults’ patients (≥ 40 years old for medical patients and ≥ 18 years old for surgical patients) of both sexes, who were admitted to any of the participating centers for serious medical illness or surgical indication, were included. We excluded patients with current or recent (1 month before the study) deep venous thrombosis or pulmonary embolism, history of intake of anticoagulant for another co-morbidity in the last month before study’s enrollment, weight below 40 kg or above 100 kg, impaired kidney or liver functions, concomitant participation to a clinical, or missing hospital chart. Pregnant or lactating women were excluded as well.

### Sample size calculation and sampling methods

The primary outcome of the present study was the percentage of patients eligible for VTE prophylaxis who are receiving appropriate prophylaxis treatment. According to the AVAIL ME study, the reported that the overall rate of VTE prophylaxis use in the Middle East among eligible patients was 47.8% for medical patients and 60% for surgical patients; while the rate of compliance to ACCP guidelines was only 36% [22]. Thus, was assumed that 40% of the eligible patients for VTE prophylaxis are actually receiving appropriate prophylaxis treatment globally. With a margin of error of 3.6% and a 95% confidence interval, we calculated that the required sample size will be 700 patients.

The present study utilized a non-probability, consecutive, sampling technique to recruit eligible patients. Investigators were asked to recruit consecutive eligible patients and each selected physician included about 10 to 20 patients who met the inclusion criteria per consulting session.

### Data collection and study’s outcomes

The following data were collected from every eligible patient: demographic characteristics, anthropometric measures (weight, height, and body mass index [BMI]), cause of hospital admission, length of hospital stay, type and duration surgery for surgical patients, risk factors for VTE, type and frequency of VTE prophylaxis, and eligibility for VTE prophylaxis according to the ACCP 2016 guidelines [23].

The primary outcome of the present study was the percentage of patients receiving appropriate VTE prophylaxis among the patients eligible for prophylaxis. The secondary outcomes were the rate of patients receiving prophylaxis treatment without being eligible for such treatment as per the guideline, the profile of the medically ill and surgical hospitalized patients and their VTE risk, the compliance rate of VTE prophylaxis in the different specialties/type of surgery, VTE prophylaxis differences between different regions and geographies, and the predictive factors or barriers for appropriate prophylaxis.

### Statistical methods

All variables recorded during the study were summarized. Frequencies and percentages (with 95% confidence interval [CI] for the primary endpoint) were provided for categorical variables. Mean and standard deviation were provided for continuous variables. The analysis was stratified by type of patient (surgical vs. medical), type of surgery, country, and specialty of the doctors. Multivariate logistic regression models were conducted using the baseline factors to assess their effect as predictors of the appropriate use of VTE prophylaxis. A *p*-value of less than 5% was considered statistically significant. All statistical tests were performed using SPSS program version 25 (IBM, Armonk, NY, USA).

## Results

The present study screened 705 patients from 40 sites across Lebanon and Jordan. Of them, 704 patients (400 from Jordan and 304 from Lebanon) (99.9%) were eligible for final analysis and one patient (0.1%) was excluded as the weight was above 100 kg. Regarding the demographic characteristics of the included patients, the patients’ age ranged from 18 to 93 years with a mean age of 54.9 ± 17.5 years. Almost 48% of the patients were males. The mean weight and height of the included patients were 75.7 ± 13.4 kg and 165.9 ± 9.3 cm, respectively. The BMI of the included patients ranged from 16.1 to 44.2 kg/m^2^ with a mean BMI of 27.5 ± 4.8 kg/m^2^. In terms of vital signs, the mean SBP and DBP of the included patients were 124.1 ± 15.4 and 73.8 ± 9.7 mmHg, respectively. Sixty percent of the patients had one or more current medical conditions. The most commonly encountered medical condition was hypertension (34.8%), followed by diabetes mellitus (22.6%) and coronary artery disease (10.1%). Two-hundred and forty-six (34.9%) patients were admitted for medical causes only, 449 (63.8%) patients were admitted for surgical causes only, and 9 (1.3%) patients were admitted for both medical and surgical causes. The average hospital stay of the included patients was 5.8 ± 8.4 days. The Demographic and clinical characteristics of the included patients were summarized in Table 1.

**Table 1:** Demographic and clinical characteristics of the participants at baseline

Variables	Patients (***N*** = 704)
**Age in years**, mean (SD)	54.96 (17.5)
**Male**, No. (%)	340 (48.3%)
**Weight in kg**, mean (SD)	75.72 (13.4)
**Height in cm,** mean (SD	165.96 (9.3)
**BMI in kg/m**^**2**^, mean (SD)	27.47 (4.8)
**Systolic blood pressure in mmHg**, mean (SD)	124.1 (15.4)
**Diastolic blood pressure in mmHg,** mean (SD)	73.75 (9.7)
**Pulse rate in beat/min**, mean (SD)	81.38 (12.5)
**Temperature in C**, mean (SD)	36.87 (0.46)
**Hypertension,** No. (%)	245 (34.8)
**Diabetes,** No. (%)	159 (22.6)
**History of other chronic illness**, No. (%) ^a^	503 (71.4)
**Cause of admission**, No. (%)	
- Medical Causes	246 (34.94%)
- Surgical Causes	449 (63.78%)
- Medical and Surgical Causes	9 (1.28%)
**Type of Surgery**, No. (%)	
- Open	327 (46.45%)
- Laparoscopic	131 (18.61%)
**Hospital Stay in days**, mean (SD)	5.83 (8.4)
**Risk factors for VTE,** No. (%)	
- Surgical risk	391 (55.5%)
- Medical risk	221 (31.4%)
- Medical and surgical risk	4 (0.6%)
**Surgical risk factors for VTE,** No. (%)	
- Obesity	237 (33.7)
- Age > 60 years old	117 (16.7%)
- Laparoscopic Surgery (> 45 Min)	59 (8.38)
- Other factors^a^	523 (74.3)
**Degree of surgical risk factors for VTE,** No. (%)	
- Low	162 (23.1)
- Moderate	109 (15.48)
- High	86 (12.22)
- Highest	38 (5.4)
**Medical risk factors for VTE,** No. (%)	
- Elderly Age (≥ 70 Years)	94 (13.35)
- Reduced Mobility (At Least 72 h)	93 (13.2)
- Obesity	86 (12.22)
- Other factors	181 (25.7%)
**Risk factors associated with increased bleeding,** No. (%)	60 (8.5)
**Risk factors associated with Mechanical Prophylaxis**	25 (3.6%)

*VTE* Venous thromboembolism, *SD* Standard deviation

^a^Patient may have more than one chronic condition

Six hundred and sixteen patients (87.5%) had one or more risk factors for VTE which were either surgical (55.5%), medical (31.4%), or surgical and medical risk factors (0.6%). Sixty patients (8.5%) had risk factors associated with increased bleeding such as active bleeding (1.1%) and low platelet count. In addition, 25 (3.5%) patients had risk factors associated with mechanical prophylaxis which were severe peripheral arterial disease (0.4%), congestive heart failure (2.8%), and acute superficial/deep vein thrombosis (0.3%).

Among the 704 patients who were eligible for the final analysis, 415 (58.9%) patients received prophylaxis treatment in form of pharmacological anticoagulant prophylaxis (*n* = 371, 52.7%), mechanical prophylaxis (*n* = 13, 1.8%), and pharmacological plus mechanical prophylaxis (*n* = 31, 4.4%). LMWH was the most commonly used anticoagulant for VTE prophylaxis (*n* = 366); however, the unfractionated heparin was administrated in 56 patients only as seen in Table 2. In Lebanon as well as Jordan, LMWH was the most commonly used anticoagulant for VTE prophylaxis (*N* = 192, 48%) and (*N* = 174, 57.2%), respectively (Table 3).

**Table 2:** VTE prophylaxis/treatment of the included patients

Variables	Patients (***N*** = 704)
**No. of patients received prophylaxis**, No. (%)	415 (58.9)
- Pharmacological anticoagulant prophylaxis	371 (52.7)
- Mechanical prophylaxis	13 (1.8)
- Pharmacological and Mechanical prophylaxis	31 (4.4)
**Types of anticoagulant**, No. (%)	
- LMWH	366 (51.9)
- Unfractionated Heparin	56 (7.9)
**Timing of anticoagulants for surgical patients**, No. (%)	
- Preoperatively	138 (59.23)
- Postoperatively	94 (40.34)
- Preoperatively/ Postoperatively	1 (0.43)
**No. of patients continued prophylaxis after discharge**, No. (%)	128 (30.84)
**Duration of LMWH after discharge**, mean (SD)	17.58 (80.9)
**Duration of Warfarin after discharge**, mean (SD)	28 (16.2)
**Concomitant treatment** No. (%)	460 (65.34)

*LMWH* Low molecular weight heparin, *SD* Standard deviation

**Table 3:** VTE prophylaxis differences according to the included countries

Variables, No (%)	Jordan (*N* = 400)	Lebanon (*N* = 304)
LMWH	192 (48.0%)	174 (57.2%)
Unfractionated Heparin	30 (7.5%)	28 (9.2%)
Other Anticoagulants	3 (0.8%)	1 (0.3%)
Mechanical Prophylaxis	12 (3.0%)	32 (10.5%)

*LMWH* Low molecular weight heparin

Among surgical patients who received anticoagulants (*N* = 233), 59.2% of them received the drug preoperatively and 40.3% received it postoperatively. Only 6.3% of the patients received mechanical prophylaxis in the form of graduated compression stockings or intermittent pneumatic compression. Almost 31% of the patients continued anticoagulants treatment after discharge in the form of LMWH (85.9%), aspirin (10.2%), warfarin (3.1%), and Fondaparinux (0.8%).

Among the total 704 eligible patients, 415 (58.9%) patients received VTE prophylaxis, while 289 (41.1%) did not receive prophylaxis. For those who received VTE prophylactic treatment, 216 (52, 95% CI [47.1–56.9%) received appropriate prophylactic agents according to ACCP guidelines. For those who were not treated with prophylactic agents, 212 (73.4, 95% CI [67.9–78.4%) were eligible for VTE prophylaxis according to ACCP guidelines as presented in Table 4.

**Table 4:** Appropriate VTE prophylaxis according to ACCP guidelines

Variables, No (%)	Eligible for prophylaxis^a^	Not Eligible for prophylaxis^a^	Total
**Received prophylaxis**	216 (30.68%)	199 (28.27%)	415 (58.95%)
**Not received prophylaxis**	212 (30.11%)	77 (10.94%)	289 (41.05%)
**Total**	428 (60.79%)	276 (39.21%)	704 (100%)

^a^According to ACCP guidelines

**Fig. 1:**
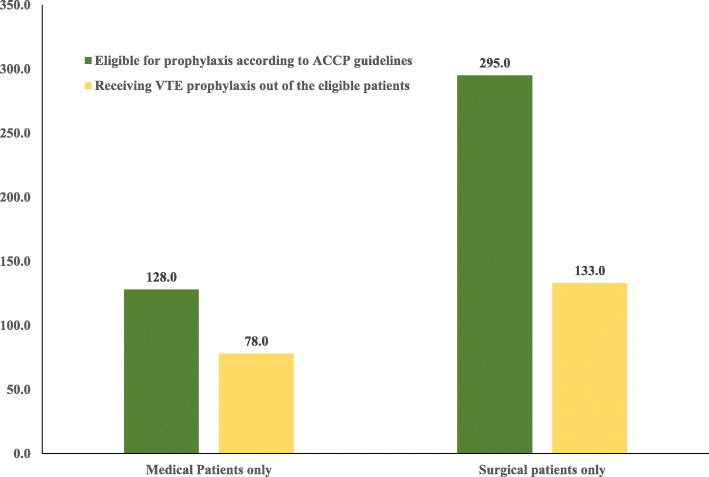
VTE prophylaxis out of the patients eligible for prophylaxis according to ACCP guidelines. ACCP; American College of Chest Physicians, VTE; venous thromboembolism

About 60.9% (95% CI 51.9–69.4%) of the patients (*n* = 78) received VTE prophylaxis out of the medical patients who were eligible for prophylaxis according to ACCP 2016 guideline (*n* = 128). While in surgical patients, only 45.1% (95% CI 39.3–51%) of the patients (*n* = 133) received VTE prophylaxis out of those who were eligible for prophylaxis (*n* = 295), Fig. 1*.* All patients with combined medical and surgical conditions received appropriate prophylaxis.

**Fig. 2:**
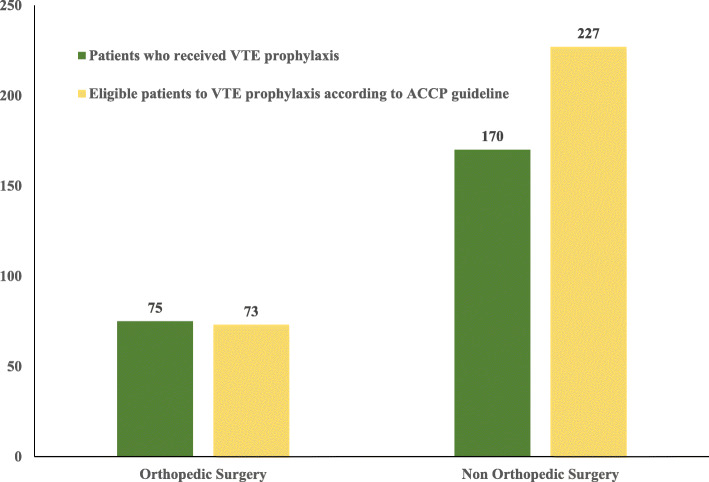
VTE prophylaxis in surgical patients out of the patients eligible for prophylaxis according to ACCP guidelines. ACCP; American College of Chest Physicians, VTE; venous thromboembolism

Regarding the orthopedic surgery, most of the patients received appropriate VTE prophylaxis according to ACCP 2016 guideline. While in non-orthopedic surgery, the number of patients who received appropriate VTE prophylaxis (*N* = 170) was lower than the number of patients who were eligible for prophylaxis (*N* = 227) as seen in Fig. 2*.*

The supplementary file no.2 shows the distribution of appropriate VTE prophylaxis according to doctors’ specialty and the type of surgery.

Overall, the rate of compliance to ACCP guidelines was higher in private hospitals than in public hospitals (85% versus 57.8%, respectively). The rate of compliance to ACCP guidelines was higher among oncologists (73.3%) and general family specialists (72%) than other specialized doctors. The rate of compliance was higher among cases of orthopedic surgeries (100%) and oncological surgeries (79%) than other types of surgery as presented in Table 5*.*

**Table 5:** Rates of Compliance to ACCP guidelines

Variables, No (%)	Compliant to ACCP guidelines	Not Compliant to ACCP guidelines	Total
**Type of hospital**			
- Private Hospital	125 (85.03)	22 (14.97)	147
- Public Hospital	168 (57.8)	22 (11.58)	190
**Risk Assessment Method implementation**	170 (37.86)	279 (62.14)	449
**Age of patients**			
- ≥ 40 years old	262 (47.4)	291 (52.6)	553
- < 40 years old	31 (20.5%)	120 (79.5%)	151
**Physician Speciality**			
- Pneumology	33 (47.14)	37 (52.86)	70
- Cardiology	32 (40.51)	47 (59.49)	79
- Internal Medicine	37 (39.78)	56 (60.22)	93
- Oncology	11 (73.33)	4 (26.67)	15
- Infectious Disease	6 (26.09)	17 (73.91)	23
- General/Family Medicine	18 (72.0)	7 (28.0)	25
- Orthopaedics Surgery	53 (42.74)	71 (57.26)	124
- Vascular Surgery	24 (60.0)	16 (40.0)	40
- General Surgery	84 (38.53)	134 (61.47)	218
- Other Surgery	26 (34.67)	49 (65.33)	75
**Type of surgery**			
- Hip replacement	16 (100.00%	0	16
- Knee replacement	19 (100.00%	0	19
- Hip fracture	21 (100.00%	0	21
- Curative arthroscopy	0 (0.00%	6 (100)	6
- Other Ortho trauma	1 (3.03%	32 (96.97)	33
- Colon /small bowel	17 (51.52%	16 (48.48)	33
- Rectosigmoid	7 (63.64%	4 (36.36)	11
- Gastric	6 (31.58%	13 (68.42)	19
- Hepatobiliary	30 (45.45%	36 (54.55)	66
- Vascular	0 (0.00%	1 (100)	1
- Thoracic	4 (28.57%	10 (71.43)	14
- Oncologic	15 (78.95%	4 (21.05)	19
- Others	60 (28.57%	150 (71.43)	210

*ACCP* American College of Chest Physicians

The multivariate logistic regression analysis showed that only age was a significant predictor of appropriate VTE prophylaxis in the present study (OR 1.05, 95% CI [1.04–1.07], *P* < 0.001) (Table 6).

**Table 6:** Multivariate logistic regression analysis to identify the significant Predictor Variables for appropriate prophylaxis

	OR	95% C.I. for OR		***P*** value
		Lower	Upper	
**Age**	1.05	1.042	1.071	**< 0.001**^a^
**Gender**	1.11	0.764	1.627	0.573
**BMI**	0.99	0.954	1.033	0.719
**SBP**	0.98	0.974	1.004	0.145
**DBP**	1.00	0.983	1.031	0.567
**HR**	1.01	0.994	1.027	0.226
**Temperature**	1.13	0.733	1.768	0.564
**Hospital Stay**	1.02	0.996	1.047	0.094
**Included hospitals**	0.97	0.918	1.041	0.480
**Type of hospital**	1.48	0.872	2.505	0.147
**Site of hospitals**	1.23	0.986	1.558	0.066
**Medical Reasons**	2.95	0.536	16.281	0.213
**Surgical Reasons**	0.68	0.125	3.771	0.664

*OR* Odds ratio, *CI* Confidence interval

^a^Patients’ age is the only significant predictor for appropriate prophylaxis

## Discussion

There is a growing body of evidence that shows substantial underuse of VTE prophylaxis in patients at risk [7]. However, the extent of VTE prophylaxis underuse in the Middle East is unclear. In the present observational, multinational, study, we found that the rates of the appropriate use of VTE prophylaxis were 60.93 and 45.08% of the medical and surgical hospitalized patients, who were eligible for VTE prophylaxis, respectively. Notably, there were wide variations in the rates of the appropriate use of VTE prophylaxis according to the type of hospitals, geographical areas, and the specialty of treating physicians. Finally, in the present study, we observed that the rate of an appropriate VTE prophylaxis according to the ACCP guidelines was increasing with the age of the patients: patients with old age (≥ 40 years) were associated with a higher rate of compliance to ACCP guidelines than young patients (< 40 years) [47.4% versus 20.5%]. In addition, the multivariate logistic regression analysis showed that only age was a significant predictor of appropriate VTE prophylaxis in the present study (OR 1.05, 95% CI [1.042–1.07], *P* < 0.001).

In 2017, Levine and colleagues showed that increasing age (OR 0.97, P < 0.001) of patients and a primary cardiovascular diagnosis (OR 0.18, P < 0.001) (chest pain, congestive heart failure, syncope/near-syncope, chronic ischemic heart disease, sinus tachycardia) decreased the likelihood of VTE prophylaxis [24].

The risk of VTE is a 10-fold higher in patients who are hospitalized after trauma, surgery, immobilizing medical illness, or pregnant and puerperal women than the general population. Accordingly, recent clinical guidelines strongly recommend the provision of pharmacological VTE prophylaxis in acutely or critically ill inpatients at risk [23, 25]. However, the present study showed that there is substantial underuse of VTE prophylaxis among hospitalized patients in Lebanon and Jordan; less than two-thirds of eligible medical patients received appropriate prophylaxis, while this rate was even much lower in surgical patients. The rate of appropriate VTE prophylaxis varied across different hospitals, geographical areas, and specialties; while the rate of compliance to ACCP guidelines was as low as 26% in some hospitals. These findings reflect the lack of standardized protocols for VTE prophylaxis use for hospitalized patients in both countries. Another possible explanation is the wide difference in the nature of participating hospitals; some of them had rural clinics and some of them were university/teaching hospitals. In accordance with our findings, a multicenter study from Lebanon in hospitalized patients reported that the rate of appropriate VTE prophylaxis was 65% of patients at low risk, 30% of patients at moderate risk, and 61% of patients at high risk [19]. A more recent report from Jordan reported that the rate of appropriate VTE prophylaxis was 67% in patients admitted to Jordan University Hospital; the study also demonstrated low compliance with the institutional guideline [26]. Other reports showed low rates of appropriate VTE prophylaxis use and guidelines compliance in Saudi Arabia [27] and Iran [28]. The AVAIL ME study reported that the overall rate of VTE prophylaxis use in the Middle East among eligible patients was 47.8% for medical patients and 60% for surgical patients; while the rate of compliance to ACCP guidelines was only 36% [22]. Globally, the multinational multicenter IMPROVE study reported that the appropriate use of VTE prophylaxis in eligible medical patients was 60% [29].

Although the underutilization of VTE prophylaxis in at-risk patients represents a major cause of in-hospital mortality and morbidity, inappropriate use of VTE prophylaxis in low-risk patients, when not medically indicated, can have a negative impact on patients’ outcomes leading to bleeding and drugs interaction [30]. In the present study, we found that 77.97% of the medical patients, who were not eligible for VTE prophylaxis, received prophylactic agents, compared to 66.88% of the surgical patients who were not eligible for VTE prophylaxis. Similar to our findings, a multicenter study from the United States (US) reported that 77.9% of hospitalized patients received excessive VTE prophylaxis without appropriate indication [31]. Another retrospective cohort study reported that pharmacological VTE prophylaxis was present in 74% of low-risk patients, who were not eligible for prophylaxis [30].

## Conclusion

*In conclusion,* the rates of the appropriate use of VTE prophylaxis among hospitalized patients and guideline compliance are low in Lebanon and Jordan. There are wide variations in the rates of the appropriate use of VTE prophylaxis according to the type of hospitals, geographical areas, and the specialty of treating physicians; therefore, awareness campaigns about the appropriate VTE prophylaxis should be performed. Besides, standardized protocols for VTE prophylaxis use for hospitalized patients in both countries should be developed and utilized. Appropriate use of VTE prophylaxis can substantially reduce the costs associated with treating VTE.

## Supplementary Information

**Additional file 1. **STROBE checklist of items that should be included in reports of cross-sectional studies.

**Additional file 2. **VTE prophylaxis differences according to doctor’s specialty and types of surgery.

## Data Availability

Not applicable.

## Abbreviations

ACCP: American College of Chest Physicians
BMI: Body mass index
CI: Confidence interval
DVT: Deep venous thrombosis
GCSs: Graduated compression stockings
IPC: Intermittent pneumatic compression
LMWH: Low molecular weight heparin
VTE: Venous thromboembolism
UFH: Unfractionated heparin
US: United States
