# Supplementary material for: A cross-sectional, multicenter, observational study to assess the prophylaxis of venous thromboembolism in Lebanese and Jordanian hospitals
Source: Thromb J. 2021 Feb 10;19:9. doi: 10.1186/s12959-021-00261-2 (PMC7877011; doi:10.1186/s12959-021-00261-2)
Supplement: Supplementary file 2 — Additional file 2. VTE prophylaxis differences according to doctor’s specialty and types of surgery. [file 12959_2021_261_MOESM2_ESM.docx]

Supplementary file 2:

VTE prophylaxis differences according to doctor’s specialty

| Doctor’s speciality | **LMWH** | | **UFH** | | **Others** | | **Mechanical** | | **Total N** |
| --- | --- | --- | --- | --- | --- | --- | --- | --- | --- |
|  | **N** | **%** | **N** | **%** | **N** | **%** | **N** | **%** |  |
| Cardiology | 38 | 77.55% | 0 | 0.00% | 0 | 0.00% | 0 | 0.00% | 49 |
| Pneumology | 40 | 57.14% | 1 | 1.43% | 0 | 0.00% | 2 | 2.86% | 70 |
| General/Family Medicine | 24 | 96.00% | 0 | 0.00% | 0 | 0.00% | 0 | 0.00% | 25 |
| Infectious Disease | 8 | 80.00% | 2 | 20.00% | 0 | 0.00% | 0 | 0.00% | 10 |
| Internal Medicine | 21 | 42.00% | 0 | 0.00% | 0 | 0.00% | 0 | 0.00% | 50 |
| Internal Medicine/Cardiology | 4 | 13.33% | 23 | 76.67% | 0 | 0.00% | 0 | 0.00% | 30 |
| Internal Medicine/Infectious Disease | 11 | 84.62% | 0 | 0.00% | 0 | 0.00% | 0 | 0.00% | 13 |
| Orthopaedics Surgery | 76 | 61.29% | 1 | 0.81% | 0 | 0.00% | 0 | 0.00% | 124 |
| General Surgery | 93 | 42.66% | 0 | 0.00% | 1 | 0.46% | 28 | 12.84% | 218 |
| Vascular Surgery | 14 | 35.00% |  | 0.00% | 0 | 0.00% | 4 | 10.00% | 40 |
| Other Surgery (cardiothoracic and Head & neck) | 37 | 49.33% | 30 | 40.00% | 3 | 4.00% | 10 | 13.33% | 75 |

| Doctor’s speciality | **Patients who received VTE prophylaxis** | | **Eligible patients to VTE prophylaxis**  **according to ACCP guidelines** | |
| --- | --- | --- | --- | --- |
|  | **N** | **%** | **N** | **%** |
| Cardiology | 38 | 77.55% | 19 | 38.78% |
| Pneumology | 42 | 60.00% | 29 | 41.43% |
| General/Family Medicine | 24 | 96.00% | 19 | 76.00% |
| Infectious Disease | 10 | 100.00% | 4 | 40.00% |
| Internal Medicine | 21 | 42.00% | 38 | 76.00% |
| Internal Medicine/Cardiology | 26 | 86.67% | 16 | 53.33% |
| Internal Medicine/Infectious Disease | 11 | 84.62% | 4 | 30.77% |
| Orthopaedics Surgery | 76 | 61.29% | 97 | 78.23% |
| General Surgery | 101 | 46.33% | 143 | 65.60% |
| Vascular Surgery | 15 | 37.50% | 15 | 37.50% |
| Other Surgery (cardiothoracic and Head&neck) | 51 | 68.00% | 44 | 58.67% |

VTE prophylaxis differences according to the types of surgery

| **Types of surgery** | **Low Molecular Weight Heparin** | | **Unfractionated Heparin** | | **Other Anticoagulants** | | **Mechanical**  **prophylaxis** | |
| --- | --- | --- | --- | --- | --- | --- | --- | --- |
|  | **Yes** | **%** | **Yes** | **%** | **Yes** | **%** | **Yes** | **%** |
| **Hip replacement** | 15 | 93.75% | 0 | 0.00% | 0 | 0.0% | 0 | 0.00% |
| **Knee replacement** | 19 | 100.00% | 0 | 0.00% | 0 | 0.0% | 0 | 0.00% |
| **Hip fracture** | 21 | 100.00% | 0 | 0.00% | 0 | 0.0% | 2 | 9.52% |
| **Curative arthroscopy** | 1 | 16.67% | 0 | 0.00% | 0 | 0.0% | 0 | 0.00% |
| **Other Ortho trauma** | 18 | 56.25% | 2 | 6.25% | 1 | 3.13% | 4 | 12.50% |
| **Colon /small bowel** | 17 | 51.52% | 0 | 0.00% | 0 | 0.0% | 10 | 30.30% |
| **Rectosigmoid** | 9 | 81.82% | 0 | 0.00% | 0 | 0.0% | 1 | 9.09% |
| **Gastric** | 14 | 73.68% | 0 | 0.00% | 0 | 0.0% | 0 | 0.00% |
| **Hepatobiliary** | 24 | 37.50% | 0 | 0.00% | 0 | 0.0% | 5 | 7.81% |
| **Urologic** | 0 | 0.0% | 0 | 0.0% | 0 | 0.0% | 0 | 0.0% |
| **Vascular** | 1 | 100.00% | 1 | 100.00% | 0 | 0.0% | 1 | 100.00% |
| **Thoracic** | 9 | 75.00% | 1 | 8.33% | 0 | 0.0% | 1 | 8.33% |
| **Oncologic** | 6 | 33.33% | 8 | 44.44% | 0 | 0.0% | 21 | 116.67% |
| **Others** | 73 | 35.44% | 20 | 9.71% | 3 | 1.46% | 0 | 0.00% |

| **Types of Surgery** | **Patients who received VTE prophylaxis** | | **Eligible patients to VTE prophylaxis**  **according to ACCP guidelines** | |
| --- | --- | --- | --- | --- |
|  | **N** | **%** | **N** | **%** |
| **Hip replacement** | 15 | 93.75% | 15 | 93.75% |
| **Knee replacement** | 19 | 100.00% | 19 | 100.00% |
| **Hip fracture** | 21 | 100.00% | 21 | 100.00% |
| **Curative arthroscopy** | 1 | 16.67% | 5 | 83.33% |
| **Other Ortho trauma** | 19 | 59.38% | 13 | 40.63% |
| **Colon /small bowel** | 17 | 51.52% | 23 | 69.70% |
| **Rectosigmoid** | 9 | 81.82% | 7 | 63.64% |
| **Gastric** | 14 | 73.68% | 7 | 36.84% |
| **Hepatobiliary** | 24 | 37.50% | 43 | 67.19% |
| **Urologic** | 0 | 0.00% | 0 | 0.00% |
| **Vascular** | 1 | 100.00% | 0 | 0.00% |
| **Thoracic** | 7 | 58.33% | 6 | 50.00% |
| **Oncologic** | 11 | 61.11% | 13 | 72.22% |
| **Others** | 87 | 42.23% | 128 | 62.14% |
